# Comparison of Clinical Characteristics and End-of-Life Care Between COVID-19 and Non-COVID-19 Muslim Patients During the 2020 Pandemic

**DOI:** 10.1177/10499091211018657

**Published:** 2021-05-27

**Authors:** Imran Khalid, Maryam Imran, Romaysaa M Yamani, Manahil Imran, Muhammad Ali Akhtar, Tabindeh Jabeen Khalid

**Affiliations:** 1 195017 Consultant King Faisal Specialist Hospital & Research Center, Jeddah & Staff Physician John D Dingell VA Medical Center, Detroit, MI, USA; 2 66787 Shifa college of Medicine, Islamabad, Pakistan; 3 195017 King Faisal Specialist Hospital & Research Center, Jeddah, Saudi Arabia

**Keywords:** Muslim, end-of-life, COVID-19, palliative care, ICU, DNAR

## Abstract

**Background:**

Little is known about end-of-life care among Muslim patients, particularly during Coronavirus disease 2019 (COVID) pandemic, which we report here.

**Methods:**

The clinical characteristics, end-of-life care and resuscitation status of Muslim patients who died in the ICU of our tertiary care hospital in year 2020 from COVID were compared to Non-COVID patients.

**Results:**

There were 32 patients in COVID and 64 in the Non-COVID group. A major proportion, mainly of Non-COVID patients, already had a hospice eligible terminal disease at baseline (p=.002). COVID patients were admitted to the ICU sooner after hospitalization (2.2 vs. 17 days), had prolonged duration of mechanical ventilation (18.5 vs. 6 days) and longer ICU stay (24 vs. 8 days) than non-COVID patients, respectively (p<.001). Almost all patients were “Full Code” initially. However, status was eventually changed to ‘do-not-attempt resuscitation’ (DNAR) in about 60% of the cohort. COVID patients were made DNAR late in their ICU stay, predominantly in the last 24 hours of life (p=.04). Until the very end, patients in both groups were on tube feeds, underwent blood draws and imaging, required high dose vasopressors, with few limitations or withdrawal of therapies. Family members were usually not present at bedside at time of death. There was minimal involvement of chaplain and palliative care services.

**Conclusions:**

Muslim COVID-19 patients had prolonged mechanical ventilation and ICU stay and a delayed decision to DNAR status than non-COVID Muslim patients. Limitation or withdrawal of therapy occurred infrequently. The utilization of chaplain and palliative care service needs improvement.

## Introduction

Coronavirus disease 2019 (COVID-19) has wreaked havoc across the globe and stretched the healthcare systems to the limits. As of April 2021, 140 million cases of COVID-19 have been documented in the world with over 3 million deaths.^1^ Many of these patients who become critically ill require ventilatory and hemodynamic support, including those who do not survive.^2^ Despite improvement in mortality with time among the COVID-19 patients admitted to the intensive care unit (ICU), still on average one out of four patients die from the disease.^3^ Most of them, however, already have multiple comorbidities which contribute to their poor outcome.^4^ Under normal circumstances, goals of care and limitations of therapy are discussed in such patients early in the hospitalization. But unfortunately, this has been deficient during the COVID-19 pandemic for obvious reasons and discussions regarding goals of care have been fewer with less utilization of palliative care services in patients admitted to the ICU.^5,6^

Muslim population in the world is estimated to be over 1.7 billion.^7^ They comprise of one out of five deaths in the world and this proportion is estimated to increase with time.^8^ However, in the United States, Muslims are still a minority population with an average estimate of 5.4 million, with sources suggesting a range of 3.4 million to 7 million.^9^ Other minority populations with COVID-19 in the United States have been found to have a disproportionate higher mortality.^10^ This has been addressed in populations like Latinos by recognizing the social inequalities, psychological challenges, spiritual and religious implications, and financial distress, along with the physical suffering when taking care of them.^11^ Unfortunately, literature reflecting on the end-of-life practices and beliefs in Muslims is scarce.^12^ The general perception from the limited data available describes Muslim patients and families being often reluctant to accept terminal diagnoses and prognoses. The acceptance of limitation or withdrawal of therapy is also believed to be less palatable to them.^12,13^ Even Muslim physicians find withdrawal of life-sustaining treatment ethically and psychologically challenging.^14^

End of life care in any patient population reflects their beliefs, culture and values and help physicians in the management plans. Despite Muslims succumbing to death from COVID-19 like other populations,^1^ there is no study that describes their end-of-life care during the pandemic. With no end in sight for the COVID-19 pandemic, it is important to understand these dynamics so physicians can integrate this information in their management strategies for the current as well as any future outbreaks. This will help address many unanswered questions regarding end-of-life care practices in Muslim patients infected with COVID-19. How aggressively are they treated? In terminal cases is there any limitation or withdrawal of therapy? When do these decisions occur in their hospitalization? Is there any acceptance of ‘do-not-attempt resuscitation’ (DNAR)? And are there any differences in these characteristics in Non-COVID Muslim patients? To answer these questions, we conducted this study and explored the clinical characteristics, end-of-life care and resuscitation status of Muslim patients who died due to COVID-19 and compared them to Muslim patients who died due to other diseases during the year 2020.

## Methods

This was a retrospective analysis and the ethical and scientific approval for the study was granted by the Institutional Review Board of the hospital.

### Study Location

The study was conducted at King Faisal Specialist Hospital & Research Center, a tertiary care teaching hospital located in Saudi Arabia, a Middle Eastern country with predominant Muslim population. The hospital is accredited by Joint Commission International and Nurses Magnet Recognition Programs. In addition to taking care of the tertiary care medical, surgical, cardiovascular, and oncological problems, the hospital runs active solid organ and bone marrow transplant programs as well. The hospital has a 26-bed medical intensive care unit (ICU). It is run by Board Certified intensivists, under a “Closed ICU” model where they are the ones primarily responsible for patient care.

### Hospital’s Patient Population

The hospital caters to the needs of tertiary care patients for the whole western region of the country, who are accepted through a referral system. Once accepted, these “eligible” patients are then followed in the hospital until they are discharged back to referring facility or succumb to their diseases. Being in a country with predominant Muslim population, Muslims comprise most of the admitted patients.^15^ All physicians who took care of these patients in ICU during study period were also Muslim, reflecting the general demographics of the country.^15^

### Study Duration and Patients

The COVID-19 outbreak started in the start of year 2020. We included all Muslim patients who were admitted to our ICU and died from COVID-19 starting January till December of 2020. Patients who died without ICU admission were excluded. Patients who died from Non-COVID causes were taken as control.

### Study Groups

Patients in the study were divided in two groups as follows:

COVID: These were the patients who were admitted to ICU with primary diagnosis of COVID pneumonia or any of the COVID-19 complications during the study period. Diagnosis for COVID-19 was made by nasopharyngeal swab using real-time polymerase chain reaction (RT-PCR).

Non-COVID: This was the control group. These were the patients who were admitted to the ICU with diagnosis other than COVID-19. COVID-19 was excluded with a negative RT-PCR. Because of the high number of Non-COVID deaths during the study period, the ratio of Non-COVID to COVID patients was taken as 2:1.

### Data Collection & Measures Compared

Patient demographics, underlying comorbidities, characteristics before and at ICU admission, clinical course in the ICU, cardiopulmonary resuscitation and DNAR details, and therapies in last 24 hours before death were recorded. These were compared between COVID and non-COVID groups. Descriptive statistics were used to organize the collected data. Continuous variables were described as mean and standard deviation (SD), and categorical variables were described as number and percentages, as appropriate. Data were analyzed using t-test or Fisher Exact test for continuous and categorical variables, respectively, with a two-sided p value of less than 0.05 considered statistically significant. Statistical analyses were performed using SAS statistical software version 9.4 (SAS Institute).

## Results

140 patients died during the study period in the ICU. 32 died from COVID-19 and were placed in the COVID group. 64 patients were taken as control and placed in the Non-COVID group. Patients in both groups had multiple comorbidities, an average of around 5, and similarly high Charlson Comorbidity Index reflective of their baseline moribund status. More than half of the patients, more so in Non-COVID group, had terminal disease at baseline which would have qualified them for hospice care before hospitalization (p=.002). This was reflected in the fact that a fair number amongst them had 2 or more hospitalizations in the year preceding death. COVID patients were older and obese than the non-COVID patients. COVID cohort comprised of more patients with solid organ transplant and hemodialysis, while non-COVID patients had higher proportion of patients with cancer or other terminal disease. [Table 1]

**Table 1.** Patient Demographics.

Parameter	COVID	Non-COVID	*p* Value
	(n=32)	(n=64)	
Age, Years, m (SD)	70 (12)	61 (16)	0.006
Gender			
Male, n (%)	23 (72)	33 (52)	0.07
Female, n (%)	9 (28)	31 (48)	
BMI, Kg/m^2^, m (SD)	32.5 (6.7)	28.6 (7.2)	0.01
Number of comorbidities, m (SD)	4.8 (1.4)	4.4 (1.4)	0.19
Charlson Comorbidity Index, m (SD)	5.2 (1.6)	5.6 (2.9)	0.46
Comorbidities, n (%)			
Hypertension	25 (78)	38 (59)	0.07
Cardiovascular disorders	19 (59)	37 (58)	0.98
Diabetes mellitus	22 (69)	34 (53)	0.18
Obesity (BMI >30 Kg/m^2^)	21 (66)	20 (31)	0.002
Hemodialysis dependent	14 (44)	9 (14)	0.002
Cancer	7 (22)	28 (44)	0.04
Chronic kidney disease	10 (31)	16 (25)	0.62
Solid organ transplant	5 (16)	1 (2)	0.01
Patients with terminal disease eligible for Hospice, n (%)	10 (31)	42 (66)	0.002
Bedridden for over a year, n (%)	3 (9)	5 (8)	0.99
>2 Hospitalizations in prior 12 months, n (%)	7 (21)	26 (41)	0.07

n: Number; m: Mean; SD: Standard deviation

COVID patients had much shorter hospital stay before ICU admission than non-COVID patients (2.2 vs 17 days), slightly lower APACHE II score (20 vs 23), lower lactic acid (2.1 vs 4.7), and were less likely to be intubated on same day of ICU admission than non-COVIDs (22% vs 59%), respectively. [Table 2] Almost all patients in both groups required invasive mechanical ventilation and on average high doses of two vasopressor agents. As opposed to short hospitalization before ICU admission, COVID patients had a prolonged ICU stay (24 vs 8 days) and a prolonged duration of mechanical ventilation (18.5 vs 6 days) than non-COVID patients, respectively. The overall hospital length of stay was about the same in both groups. ARDS was the leading cause of death amongst COVID, while septic shock was in the non-COVID cohort (p<.001).

**Table 2.** ICU Course of the Patients.

Parameter	COVID (n=32)	Non-COVID (n=64)	*p* value
Hospital length of stay prior to ICU admission, days, m (SD)	2.2 (0.9)*	17 (16)*	<.001
Patient intubated on day 1 of ICU admission, n (%)	7 (22)	38 (59)	<.001
APACHE II score on ICU admission, m (SD)	20 (6.7)	23 (6.9)	.045
Lactic acid on ICU admission, m (SD)	2.1 (1.4)	4.7 (4.1)	<.001
Required invasive mechanical ventilation, n (%)	32 (100)	61 (95)	.69
Duration of mechanical ventilation, days, m (SD)	18.5 (15)	6.3 (6.8)	<.001
Required vasopressor agents, n (%)	31 (97)	54 (84)	.06
Number of vasopressor agents, m (SD)	2.1 (0.82)	2 (0.93)	.6
Duration of vasopressor agents, days, m (SD)	4.4 (2.4)	3.7 (2.7)	.21
Patient required Dialysis, n (%)	18 (56)	27 (42)	.19
Last Lactic acid before death, m (SD)	11.1 (9.3)	12 (9.4)	.65
Cause of Death, n (%)			
ARDS/Pneumonia	16 (50)	9 (14)	<.001
Septic Shock	6 (19)	34 (53)	.001
Other Shock	4 (12)	5 (8)	.47
Other	6 (19)	16 (25)	.61
ICU length of stay, days, m (SD)	24 (18.4)	8 (7.9)	<.001
Hospital length of stay, days, m (SD)	26 (18.5)	19 (16.3)	.06

n: Number; m: Mean; SD: Standard deviation; ICU: Intensive care unit; *COVID n=16; Non-COVID n=37

Table 3 gives the detailed overview of Cardiopulmonary resuscitation and DNAR characteristics of both groups. Patients were “Full Code” at ICU admission, however, in about 60% the resuscitation status was changed to DNAR before death. Amongst those who had CPR done, there was no difference between the groups except that non-COVID patients had slightly prolonged duration of CPR. Amongst the DNAR characteristics, COVID patients had higher proportion of patients who were made DNAR after at least one cardiopulmonary arrest (16% vs 5%), were more likely to be made DNAR closer to death (2.5 vs 4 days) and had more patients made DNAR in last 24 hours of life (56% vs 23%) than Non-COVID patients, respectively (p=.04). [Table 3] DNAR discussions were driven predominantly by ICU physicians, and major decision makers for patients were usually their children in both groups. In person visitation occurred more in non-COVID patients whereas COVID patients had relatively more videoconferences by the family members. Unfortunately, very few family members were at patients’ bedsides at time of death. The involvement of chaplain and palliative care teams were minimal in the ICU.

**Table 3.** Cardiopulmonary Resuscitation and DNAR Details in ICU.

Parameter	COVID (n=32)	Non-COVID (n=64)	*p* value
Code status at ICU admission, n (%)			
Full Code	32 (100)	59 (92)	.17
DNAR	0	5 (8)	
Code status at death in ICU, n (%)			
Full Code	14 (44)	24 (38)	.65
DNAR	18 (56)	40 (62)	
CPR done in ICU, n (%)	19 (59)	26 (41)	.09
Duration of CPR, minutes, m (SD)*	15 (5.7)	20.9 (3.9)	<.001
Patient made DNAR after one CPR, n (%)^†^	5 (16)	2 (5)	.04
DNAR decision made days after admission, m (SD) ^†^	22 (14.8)	16.7 (13.9)	.19
DNAR decision made days before death, m (SD) ^†^	2.5 (2.2)	4 (2.7)	.04
Patients made DNAR in 24 hours before death, n (%)^†^	10 (56)	8 (20)	.04
DNAR documentation as per hospital policy, n (%)^†^	16 (89)	33 (83)	.70
DNAR discussion led by, n (%)^†^			.29
Intensivist	18 (100)	36 (90)	
Primary Physician	0	4 (10)	
Family members participated in DNAR discussion, n (%)^†^			
Spouse	4 (22)	7 (18)	.99
Children	8 (44)	18 (45)	1
Parent	1 (6)	2 (5)	1
Sibling	2 (11)	4 (10)	1
Others	3 (17)	9 (22)	.74
Family visited patient during hospitalization, n (%)	9 (26)	33 (52)	.03
Family saw patient via videoconference, n (%)	12 (38)	8 (13)	.007
Family at patient’s bedside at time of death, n (%)	3 (9)	13 (20)	.24
Involvement of chaplain	5 (16)	7 (11)	.52
Involvement of palliative care team	1 (3)	4 (6)	.66

* Number in COVID patients=19, Non-COVID=26; †Number COVID=18, Non-COVID=40; CPR: Cardiopulmonary resuscitation; DNAR: Do not attempt resuscitation; ICU: Intensive care unit; n: Number; m: Mean; SD: Standard deviation

In the last 24 hours before death, patients in both groups were predominantly on tube feeding, had foley catheter, had bloods drawn and imaging done, were on high doses of two vasopressors on average with majority on at least maximal dose of one vasopressor agent, few had limitations on vasopressor dosages or any withdrawal of therapy, and were intubated with an average Richmond agitation-sedation scale score of -4. COVID patients were more likely to be sedated and paralyzed than non-COVID patients. [Table 4]

**Table 4.** Therapies in last 24 Hours Before Death.

Parameter	COVID (n=32)	Non-COVID (n=64)	*p* value
Foley catheter, n (%)	18 (56)	43 (67)	.37
Tube feeding, n (%)	26 (81)	54 (84)	.77
Blood draw, n (%)	29 (91)	57 (89)	.98
Imaging, n (%)	27 (84)	44 (69)	.14
Number of vasopressors, m (SD)	2.1 (0.82)	2 (0.93)	.61
Maximal dose of at least one vasopressor, n (%)	24 (75)	37 (58)	.12
Limitation of vasopressors, n (%)	5 (16)	13 (20)	.78
Withdrawal of vasopressors, n (%)	0	6 (9)	.17
Mechanical ventilation, n (%)	32 (100)	59 (92)	.16
Sedation, n (%)	28 (88)	31 (48)	<.001
RASS score in sedated, m (SD)	-4.4 (1.2)	-4.2 (1.2)	.44
Paralytics, n (%)	13 (41)	4 (6)	<.001
Dialysis, n (%)	15 (47)	13 (20)	.009

n: Number; m: Mean; SD: Standard deviation; RASS: Richmond agitation-sedation scale

## Discussion

Our study gives a detailed insight into the end-of-life course, therapies, resuscitation decisions and clinical care of Muslim patients who died during the 2020 COVID-19 pandemic. The direct comparison between patients dying from COVID-19 and those from non-COVID conditions also highlights some insightful facts regarding the approach of the physicians towards these Muslim patients. Preferences at end of life differ among different populations.^16^ Our study about Muslim patients fills in some of the knowledge gaps in this regard.

Our patient population comprised of tertiary care patients with multiple comorbidities and had higher expected mortality. A large proportion of these patients, and more so in Non-COVID group, had illnesses that would have qualified them to be eligible for hospice care before their hospitalization. Moreover, a fair number in both groups had two or more hospitalizations in the preceding year. This lack of enrollment in hospice and the resultant recurrent admissions could be due to the reluctance of physicians in recommending hospice over active treatment or resistance from the patients or families themselves.^14^ This is definitely an area of focus that warrants deeper analyses and pragmatic solutions.

COVID-19 patients in our cohort were older and obese which is in line to what has been reported in literature.^2^ The typical initial single organ pulmonary involvement of the COVID-19 patients resulted in their rapid respiratory deterioration, shorter time to ICU admission from presentation, relatively lesser APACHE II score and normal lactic acid as compared to Non-COVID patients. However, they had prolonged duration of invasive mechanical ventilation and longer ICU stay. The major cause of death was ARDS in COVID and septic shock in Non-COVID patients, but all eventually had multiorgan failure and sever hemodynamic compromise at death. These characteristics represent typical consequences of an acute pneumonic insult in COVID-19 patients and septic shock in Non-COVID patients.

Cardiopulmonary resuscitation and DNAR details between the groups had some interesting aspects. Even though almost all patients were admitted to ICU as “full code”, 60% died without resuscitation with DNAR orders. This dispels the perception that Muslim patients or their families are adamantly opposed to the idea of no resuscitation.^12^ Some studies have explored Muslim physician perspectives regarding DNAR, but hardly any studies describe the prevalence of patients dying without resuscitation in a Muslim cohort.^14^ The key difference between the groups in our study was that COVID patients were more likely to be made DNAR later in their disease course and close to eventual death. This may reflect the hesitation on the physician’s part to give up early on these patients as reported in similar cases of acute illnesses or rapid deterioration.^17^ The DNAR documentation was completed in about 80% of the cases, which needs improvement. Amongst the family members who were the main participants in DNAR discussions, majority were children. We could not delineate whether this was due to unavailability of spouse as “next of kin” or due to sociocultural influences. This aspect of decision making amongst families of Muslim patients has not been explored before and needs more evaluation.

Due to the pandemic, restrictions were almost universally placed in terms of families visiting their loved ones admitted to the hospital, even more so, if they were infected with COVID-19.^18,19^ Our hospital was no exception and had similar policies. This resulted in fewer family meetings occurring in-person. Decreased family contact and lack of in person family meetings may contribute to poor communication and may result in fewer changes in goals of care for patients than in-person meetings.^18^ This needs to be further explored in Muslims and compared to non-pandemic situation.

The involvement of palliative care service and Muslim chaplain was very negligible in our patients. There is low utilization of palliative care services reported in patients with COVID-19 in underserved populations.^10,20^ In Muslim countries, optimal utilization of specialized spiritual and palliative care has been historically lacking and our study reflects the same findings that the deficiency still exists during the COVID pandemic.^21^ These vital aspects of supportive, spiritual, and palliative care desperately need to be improved among Muslim patient populations as they have shown to exert a positive impact.^22^

The therapies received in the last 24 hours in both groups were almost similar. Most of our patients were on tube feeding. This has been reported in other studies also where withholding nutrition is usually not accepted by Muslim families and physicians.^23^ Physicians were reluctant to withhold or withdraw vasopressors, and majority of patients in both groups ended up having labs drawn and imaging done even in last 24 hours before death. Factors influencing these practices may reflect the hesitancy on part of the Muslim physicians and have been referenced in some studies.^14,23^ Whether this practice can be changed by help of palliative care teams in Muslim patients remains to be answered.

### Limitations

Our study has a few limitations and touches on many aspects of end-of-life care dynamics in Muslim patients that need to be explored further. The pandemic times are marked with difficulty in communication in general for all groups and population, both hospitalized as well as in ICU, and results may be different for non-pandemic scenarios. It is a single center retrospective study of a single ethnic population and results may differ in other ethnicities. The cohort of 96 patients, even though is one of the largest reported, but still needs to be expanded in future studies. Many of our patients would have been suitable for hospice care before hospital admission. Whether outpatient involvement of palliative care and hospice teams would change this scenario needs to be studied. DNAR in our cohort occurred late in the disease course and impact of early palliative care involvement on end-of-life preferences needs to be evaluated. We could not identify cases where DNAR discussions were held by the physicians but failed. Prospective studies would be helpful in identifying the success of these attempts. The reluctance of physicians in withdrawal or withholding of therapy in the last few days of life also needs further evaluation. Our study is a retrospective study; therefore, it is difficult to determine causal effects for the outcomes described.

## Conclusion

Muslim COVID-19 patients have prolonged mechanical ventilation and ICU stay than non-COVID patients.Code status of over half of the patients in both groups was changed to DNAR, albeit the decision occurring late and closer to death in COVID-19 patients. Main decision makers regarding DNAR seem to be children of the critically ill patients. The practice of withholding or withdrawal of therapy at end of life is still not that prevalent. During critical times, and particularly at the end of life, upstream Palliative Care and Chaplaincy services, beyond facilitating goals of care and advanced care planning discussions, may help with supporting Muslim patients, their families, and the healthcare teams caring for them. This needs further exploration in Muslim patients and especially during a pandemic.
